# Optical coherence tomography holds promise to transform the diagnostic anatomic pathology gross evaluation process

**DOI:** 10.1117/1.JBO.27.9.096003

**Published:** 2022-09-01

**Authors:** Diana Mojahed, Matthew B. Applegate, Hua Guo, Bret Taback, Richard Ha, Hanina Hibshoosh, Christine P. Hendon

**Affiliations:** a Columbia University, Department of Biomedical Engineering, New York, United States; b Columbia University, Department of Electrical Engineering, New York, United States; c Boston University, Department of Biomedical Engineering, Boston, Massachusetts, United States; d Columbia University Irving Medical Center, Department of Pathology, New York, United States; e Columbia University Irving Medical Center, Department of Surgery, New York, United States; f Columbia University Irving Medical Center, Department of Radiology, New York, United States

**Keywords:** optical coherence tomography, pathology, breast cancer, prostate cancer, lung disease, pancreatic disease

## Abstract

**Significance:**

Real-time histology can close a variety of gaps in tissue diagnostics. Currently, gross pathology analysis of excised tissue is dependent upon visual inspection and palpation to identify regions of interest for histopathological processing. Such analysis is limited by the variable correlation between macroscopic and microscopic findings. The current standard of care is costly, burdensome, and inefficient.

**Aim:**

We are the first to address this gap by introducing optical coherence tomography (OCT) to be integrated in real-time during the pathology grossing process.

**Approach:**

This is achieved by our high-resolution, ultrahigh-speed, large field-of-view OCT device designed for this clinical application.

**Results:**

We demonstrate the feasibility of imaging tissue sections from multiple human organs (breast, prostate, lung, and pancreas) in a clinical gross pathology setting without interrupting standard workflows.

**Conclusions:**

OCT-based real-time histology evaluation holds promise for addressing a gap that has been present for >100  years.

## 1 Introduction

Technologies allowing for real-time histology evaluation may impact the entire anatomic diagnostic process across multiple organs—from biopsy to intraoperative margin assessment to tissue grossing as well as tissue banking.^1^^,^^2^ The macroscopic examination of excised tissue (i.e., tissue grossing) is a critical step in the diagnostic anatomic pathology process as it defines regions of interest requiring further analysis, but it is limited by the variable correlation between gross and microscopic findings.^3^^,^^4^ This fundamental discordance is a gap that could be closed by the application of real-time histology evaluation by optical coherence tomography (OCT). OCT is a nondestructive, 3D microscopic imaging modality with cellular-level resolution^5^ that is being applied currently in clinical ophthalmology^6^ and dermatology^7^ and is emerging in other specialties.^8^^,^^9^ OCT provides a resolution and depth of field that is midway between traditional microscopy and clinical ultrasound. Although ultrasound can achieve a large imaging depth (several centimeters), it has limited resolution (>10  μm). On the other hand, traditional microscopy techniques such as confocal microscopy have very high resolution (<1  μm), but the penetration depth is minimal (<1  mm). OCT bridges the gap between clinical and microscopic imaging, providing 1 to 2 mm of penetration depth and high resolution (1 to 10  μm). Once integrated into the diagnostic workflow, we envision OCT improving the diagnostic process for radiologists by real-time assessment of biopsies; for surgeons by intraoperative margin determination; and for pathologists by identification of regions of interest in pathology grossing/tissue banking, increasing efficiencies of the laboratory by reducing block submission, and reducing pathologist workload by reviewing fewer slides. The aim of OCT in the diagnostic arena is to provide for real-time broad-spectrum assessment of tissue characterization including normal, abnormal, and neoplastic tissues.

Although each organ and disease has a specific set of grossing guidelines, there are general principles that are employed to characterize pathology in an excised specimen: the tissue is sent to the gross room where it is first oriented, margins are inked, and the specimen is serially sectioned into 3- to 5-mm thick slices. The slices are grossly examined by visual inspection and palpation (and sometimes radiographically) to determine regions of interest. Additional random regions are examined to address the substantial problem of gross-microscopic discordance to increase the sensitivity of detection of disease at the cost of specificity.^10^^–^^14^ The process further includes placing tissue sections into tissue cassettes, followed by block generation (tissue processing and embedding), sectioning, slide staining, and review by a pathologist. The process is inefficient, laborious, and costly due to current limitations in gross tissue evaluation, both for the laboratory and pathologist. In many settings, a substantial portion of cassettes contains no useful diagnostic information. OCT imaging of gross tissue could guide region of interest definition, resulting in decreased block submission, gross room work, histology laboratory processing costs, and pathologist review time while maintaining if not increasing accuracy. This is of particular relevance given increasing gross room and histology laboratory workloads (due to increased screening/aging population), shortages in technicians, budgetary constraints, and pathologist caseloads.^3^^,^^15^

In this study, we are the first to address the gross pathology gap between macroscopic and microscopic evaluation of tissues by the introduction of OCT to be integrated in real-time during the clinical diagnostic pathology grossing process. This is achieved using a high-resolution, ultrahigh-speed, large field of view OCT device designed to meet the Functional Requirements of the College of American Pathologists (CAP) *In Vivo* Microscopy Committee (IVM). Within this paper, we demonstrate the feasibility of imaging tissue sections from multiple human organs (breast, prostate, lung, and pancreas) in a fast-paced clinical gross pathology setting.

## 2 Materials and Methods

### 2.1 Pathology OCT Scanner

**Fig. 1:**
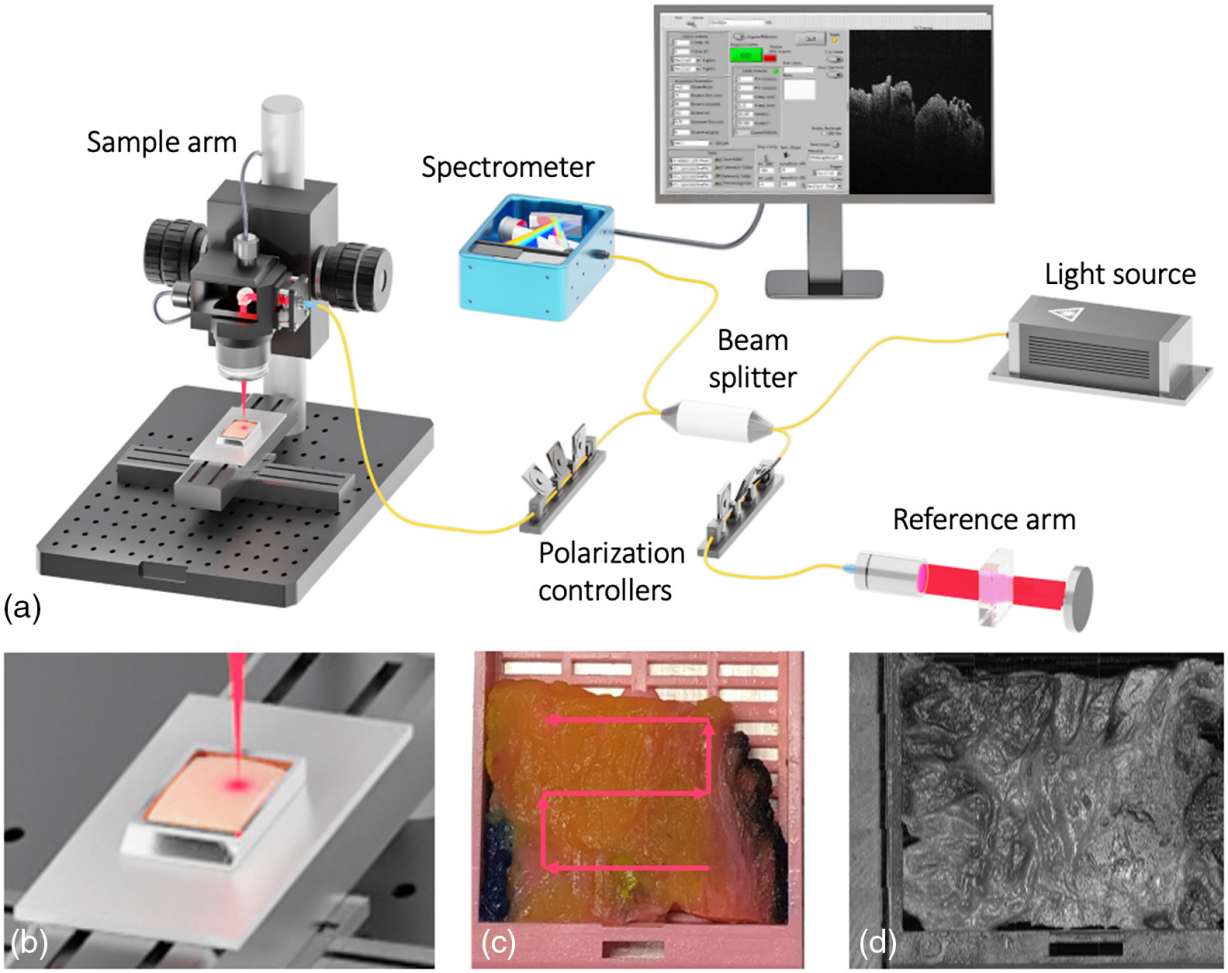
Schematic of the pathology imaging system. (a) UHS–OCT interferometer splits light from the broadband light source into the sample and reference arms and then relays light back to the spectrometer. Imaging is controlled by a GUI, which allows for rapid visualization and processing of OCT images. (b) Zoomed-in view of tissue block in pathology cassette in cassette holder. (c) Snake-scan imaging pattern allows the full specimen to be imaged. (d) Resulting stitched OCT represented in an *en face* projection that shows entire tissue surface structure. Scale bar=3  mm.

The imaging device used in this study is a custom-designed system based on spectral-domain OCT [Fig. 1(a)]. The light source was a multiplexed superluminescent diode (IPSDW0825, InPhenix) with a center wavelength of 850 nm and a bandwidth of 100 nm (coherence length of 7.25  μm). In the fiber-based OCT system, a 50/50 fiber optic coupler (TW850R2A2, Thorlabs) was used to separate the light into the sample and reference arms. Two triplet collimators (TC12APC-780, Thorlabs) were used to collimate the light in both arms. Polarization controllers (FPC030, Thorlabs) maintained the polarization state of both arms. In the sample arm, a two-axis galvanometer scanner (GVS002, Thorlabs) was used to scan the beam prompted by a digital-to-analog connector block (BNC-2110, National Instruments) controlled by a data acquisition (DAQ) card (PCI-6255, National Instruments). A low-NA scan lens (NA=0.055, LSM03-BB, Thorlabs) was used as the imaging lens. In the reference arm, a glass block was used for dispersion compensation (LSM03DC, Thorlabs). The reflected light was relayed to a high-speed spectrometer (Cobra-S 800, Wasatch Photonics). The spectrometer detected a spectral range covering 750 to 930 nm and acquired A-lines at a rate of 250 kHz with 1024 A-lines per B-scan. Each B-scan was composed of multiple A-lines acquired across a transverse plane. The imaging range of the spectrometer was 3.00 mm. The 2048-pixel camera (OctoPlus, E2V) was controlled by a CameraLink framegrabber (PCIe-1437, National Instruments). The reference signal was acquired using a diaphragm shutter (SHB1T, Thorlabs) that is controlled by the digital-to-analog connector block.

**Fig. 2:**
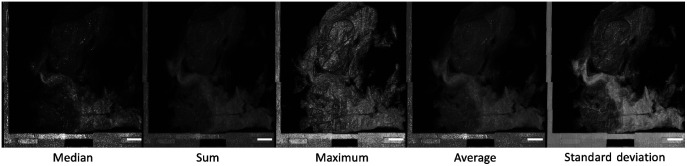
Different types of intensity projections along the depth (z-axis) of the tissue. Median, sum, maximum, average, and standard deviation projections highlight different features of this breast tissue with cystic change and IDC. The maximum projection shows the external features of the tissue, whereas other projection operations are more suited for examining the internal features of the tissue. The maximum shows a clear topological architecture, whereas standard deviation, median, and average show clearer tissue contrast. Scalebar=3  mm.

Our system scans across an area of up to 10  cm×10  cm using a motorized two-dimensional stage that automatically moves across the region of interest and stitches together sequential volumetric datasets (DDS100, Thorlabs). The stages were actuated by DC motor controllers (KBD101, Thorlabs). A customized cassette holder was machined from aluminum to place the tissue cassettes on the 2D scanning stage [Fig. 1(b)]. Tissue samples were placed on the lower right corner of the cassette, and the software was calibrated to begin snake-scanning from that corner [Figs. 1(c) and 1(d)]. The large field of view enabled by our system allowed us to visualize structures at both the macroscopic and microscopic levels. The user can operate the system by placing the tissue cassette in a holder on the 2D stage. The user-friendly acquisition software snake-scans the entire surface of the tissue. A schematic of the system is shown in Fig. 1. Based on the 250 kHz A-line rate, optical imaging of a typically-sized (20  mm×20  mm) pathology tissue cassette takes 40 s. Image quality only suffers minorly as the imaging speed increases (Fig. S1 in the Supplemental Material). We showcase our imaging results in different planes. The macroscopic visualization of the 3D data is visualized using Z-projections. An intensity projection along the Z-axis enables analysis of a stack by “flattening” it into a 2D representation (Fig. 2). Individual features can be seen in the Z-projections and directly compared with H&E histology. The cross-sectional features of the tissue can be seen in the B-scans, which provide further information on the tissue architecture.

The system achieves 3.7-μm axial resolution in tissue (5.52  μm in the air), 3.7-μm lateral resolution in tissue (5.52  μm in air), and 1.1 mm 6-dB sensitivity fall-off. The power on the sample is 2 mW, the images are not averaged, and the average SNR is 95 dB at 250 kHz.

The clinical-grade system is easily transportable and compact and was engineered to be easy to use in the pathology clinical setting. The imaging device, accompanying optics, electronic controllers, DAQ system, and computer were fitted on a custom medical device cart (AFC Industries) (Fig. S3 in the Supplemental Material).

### 2.2 OCT Software

We designed an acquisition software in Labview (National Instruments) that was implemented in a state machine to efficiently enable the application to perform a variety of complex tasks. Figure S2 in the Supplemental Material shows the graphical user interface (GUI). The user can adjust the acquisition parameters including A-lines/B-scan, B-scans/s (live view), B-scans/s (acquisition mode), B-scans/volume, camera exposure time, number of B-scans to average, and surface area scanned per volume through the galvanometer controls (X and Y voltage amplitudes). Dispersion compensation and spectral-shaping can be turned on and off. When imaging large areas, the user can input the X and Y dimensions to the snake-scan. The user inputs the lateral size (x,y) of the sample, and the motorized stage moves and acquires subsequent volumes in a snake-scan pattern across the sample with a small overlap between samples. There was no Z-translation; each of the volumes was automatically taken; the spacing between volumes was known and input into the acquisition parameters; and there was no overlap between volumes, so blending was not needed.

The OCT processing software was developed in the C programming language. The image processing sequence includes five steps: reference light intensity background subtraction, linear-k interpolation, apodization, digital dispersion compensation, and Fourier transform. The method for calibrating the vectors for k-linearization and dispersion compensation has been previously described.^16^^–^^18^ The average processing time is 0.08  s/B-scan.

When imaging large areas, the user can input the X and Y dimensions to the snake-scan (Fig. 1). The data are subsequently stitched in MATLAB.

### 2.3 OCT Imaging within the Anatomic Pathology Standard of Care

In Table 1, we illustrate how our ultra-high speed-OCT (UHS-OCT) system meets the CAP IVM functional requirements. The requirements include (1) a portable imaging system; (2) simple (one-step) specimen preparation that must not have adverse effects on formalin-fixed paraffin-embedded (FFPE) histology, immunohistochemistry (IHC), *in situ* hybridization (ISH), or genomic testing; (3) an imaging time of under 5 min; (4) a 1 to $5\;{\mathrm{cm}}^{2}$ imaging field of view; (5) cellular-level resolution; and (6) easy use such that a technician would be able to operate the device without trouble.

**Table 1:** How we meet CAP IVM requirements. CAP IVM functional requirements for pathology applications of *ex vivo* microscopy (EVM).^19^

Parameter	CAP IVM functional requirement	Our OCT system
Size	Must be portable	Fits on $2^{\prime }\times 2^{\prime }$ device cart
Specimen preparation	<5 min; simple, able to be performed consistently with minimal training; no adverse effect on FFPE histology, IHC, ISH, genomic testing, etc.	No specimen preparation
		Place cassette in cassette holder on motorized stage
Imaging time	No more than 1–5 min	40-s cassette imaging @ 250 kHz A-line rate
Field of view	Up to $1\text{–}5\;{\mathrm{cm}}^{2}$	10×10 cm
Diagnostic capability	Cellular resolution and ability to assess tissue types, identify lesional tissue, and assess cellularity and viability	Axial resolution: 3.7 μm (tissue)
		Lateral resolution: 3.7 μm (tissue)
Ease of use	Easy; histotechnologist or cytotechnologist should be able to operate without trouble	Easy-to-use GUI

**Fig. 3:**
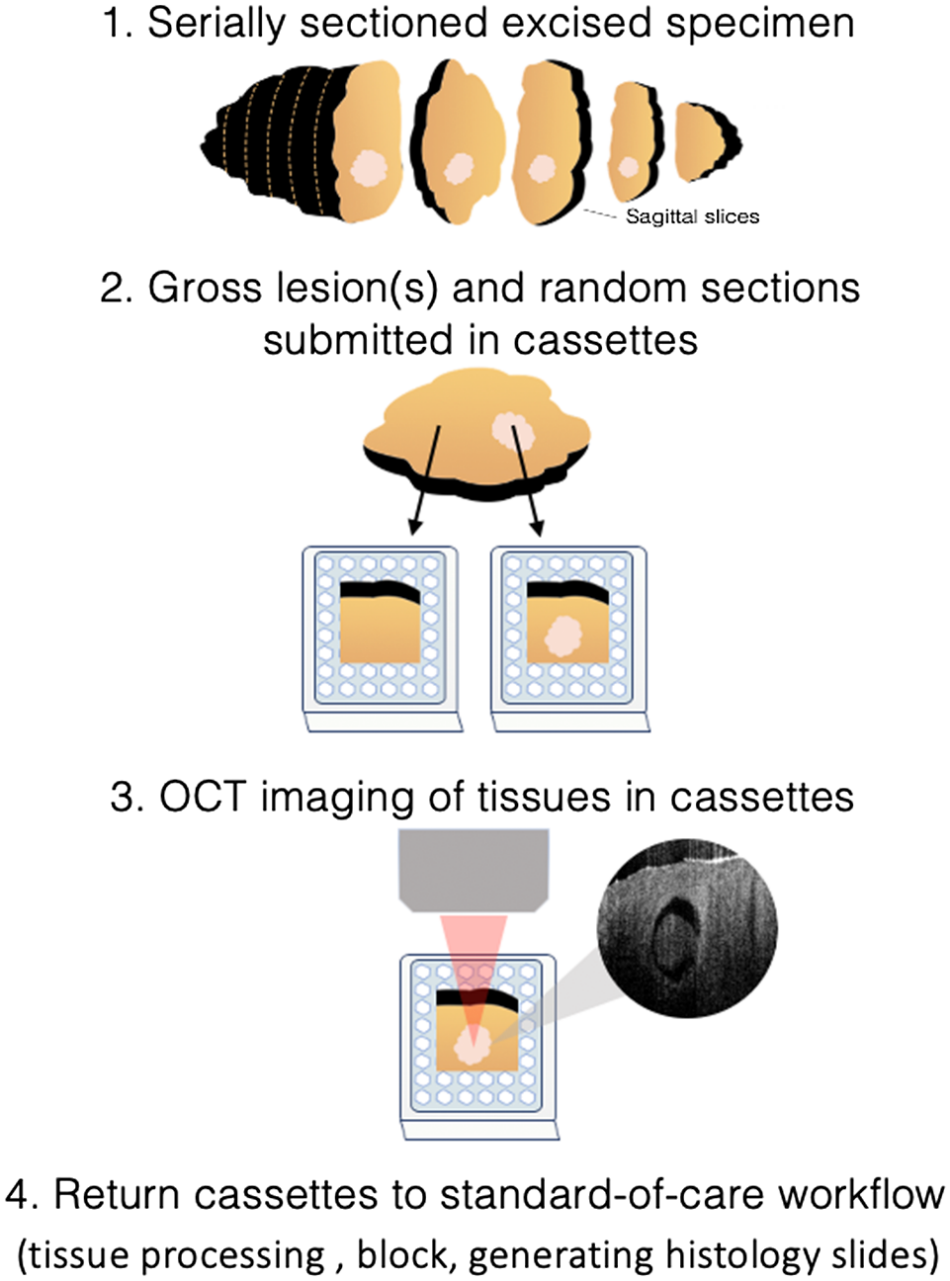
OCT imaging within the pathology workflow. After surgical specimens are serially sectioned by physician assistants, they grossly identify blocks via visual inspection and palpation. OCT imaging of the block allows for real-time histology evaluation and determination of regions of interest.

We performed all imaging in the Division of Anatomic Pathology at Columbia University Irving Medical Center. This study was approved by Columbia University Institutional Review Board (IRB) AAAS6938. We examined specimens from seven patients: two patients who underwent breast excisions (18 cassettes), two patients who underwent prostate excisions (eight cassettes), two patients who underwent lung excisions (six cassettes), and one patient who underwent pancreatic excision (four cassettes). Following surgical excision, the resected specimens were processed as per standard of care as outlined by the College of American Pathologists. Different organs have specific grossing recommendations. For instance, breast tissue requires submission of all gross lesions, random sections, and margins. Prostate tissue requires sections taken from apical anterior and posterior bilateral regions as well as seminal vesicles. The lung requires submission of mass lesions nearest pleural surfaces, adjacent lung parenchyma, bronchial margins, and lymph nodes. The pancreas requires submission of mass lesions and anterior surface and posterior margin, pancreatic and bile duct margin, lymph nodes, and duodenum (if applicable). However, they broadly followed this general protocol: slices were grossly inspected via visual inspection and palpation. In keeping with standard pathology workflow, tissues representing areas of interest and additional random sections were placed into cassettes (number and type dependent on organ). Cassettes were fixed in 10% formalin for 6 to 72 h (occasionally fixation followed serial slice sectioning). Within this fixation window, OCT imaging of tissues in cassettes was performed. Figure 3 illustrates the protocol for imaging. Following specimen orientation, inking, and serial sectioning into 3- to 5-mm slices, the physician assistants selected and submitted tissue into cassettes per standard of care. We imaged tissue samples in cassettes after they were formalin-fixed for a minimum of 6 h and a maximum of 72 h.

### 2.4 Pathology Examination

After OCT imaging, the tissues were processed and embedded into paraffin blocks, 4-μm tissue sections were cut and stained, and H&E slides were reviewed by a pathologist. The pathology slide characterization was done by a pathologist without knowledge or view of the OCT images. This study did not alter the standard of care process of sampling, we evaluated samples by OCT as well as histopathology once chosen by the grosser.

The histological processing of breast specimens followed standard clinical procedures and was not altered for the OCT imaging study. All surgical specimens were evaluated by pathologists (H.H., H.G.) based on the guidelines of the College of American Pathologists. All key histologic features were identified. *In situ* and invasive carcinomas were graded and, when appropriate, staged, and margins were determined.

Photographs of the tissue blocks were taken prior to OCT imaging and tissue processing. Corresponding OCT volumes of the blocks were captured, H&E images of the tissue blocks were digitized, and all three were commonly oriented and juxtaposed. H&E slides were reviewed for interesting histologic features, and corresponding regions were identified in OCT.

## 3 Results

### 3.1 Imaging Breast Tissues

The following are various lesions of the breast as imaged by OCT and compared with H&E histology. These include normal tissue, non-neoplastic, and neoplastic changes of the breast.

**Fig. 4:**
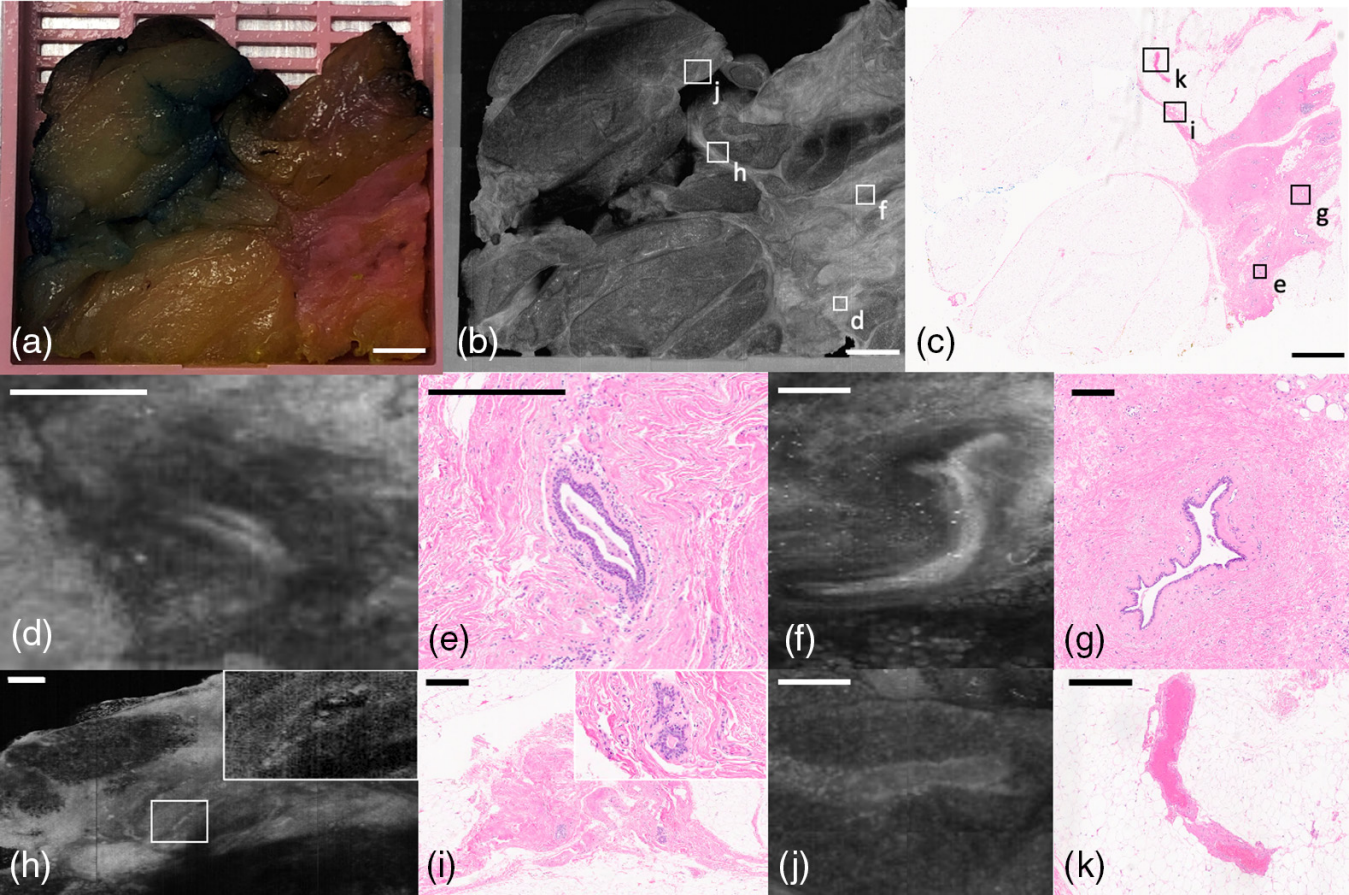
BREAST lumpectomy block containing normal breast parenchyma. (a) Photograph of specimen. (b) Average projection along z-axis. (c) H&E histology slide of block. (d) OCT *en-face* representation of mildly dilated duct; (e) corresponding H&E histology. (f) OCT *en-face* representation of fibroadenomatoid hyperplasia; (g) corresponding H&E histology. (h) OCT *en-face* representation of fibrotic region of breast with regions of collagen surrounded by adipose tissue. Inset represents breast acini that are a part of a lobule; (i) corresponding H&E histology. (j) OCT *en-face* representation of blood vessel; (k) corresponding H&E histology. (a)–(c) Scale bar=3  mm; (d)–(k) 500  μm.

OCT imaging of a prototypical example of normal breast tissue includes fibroadipose tissue, ducts, lobules (breast acini), and blood vessels (Fig. 4). The capacity to reliably identify normal tissue can be utilized to exclude areas with these features from tissue processing, enhancing the efficiency of the breast diagnostic pathology process.

**Fig. 5:**
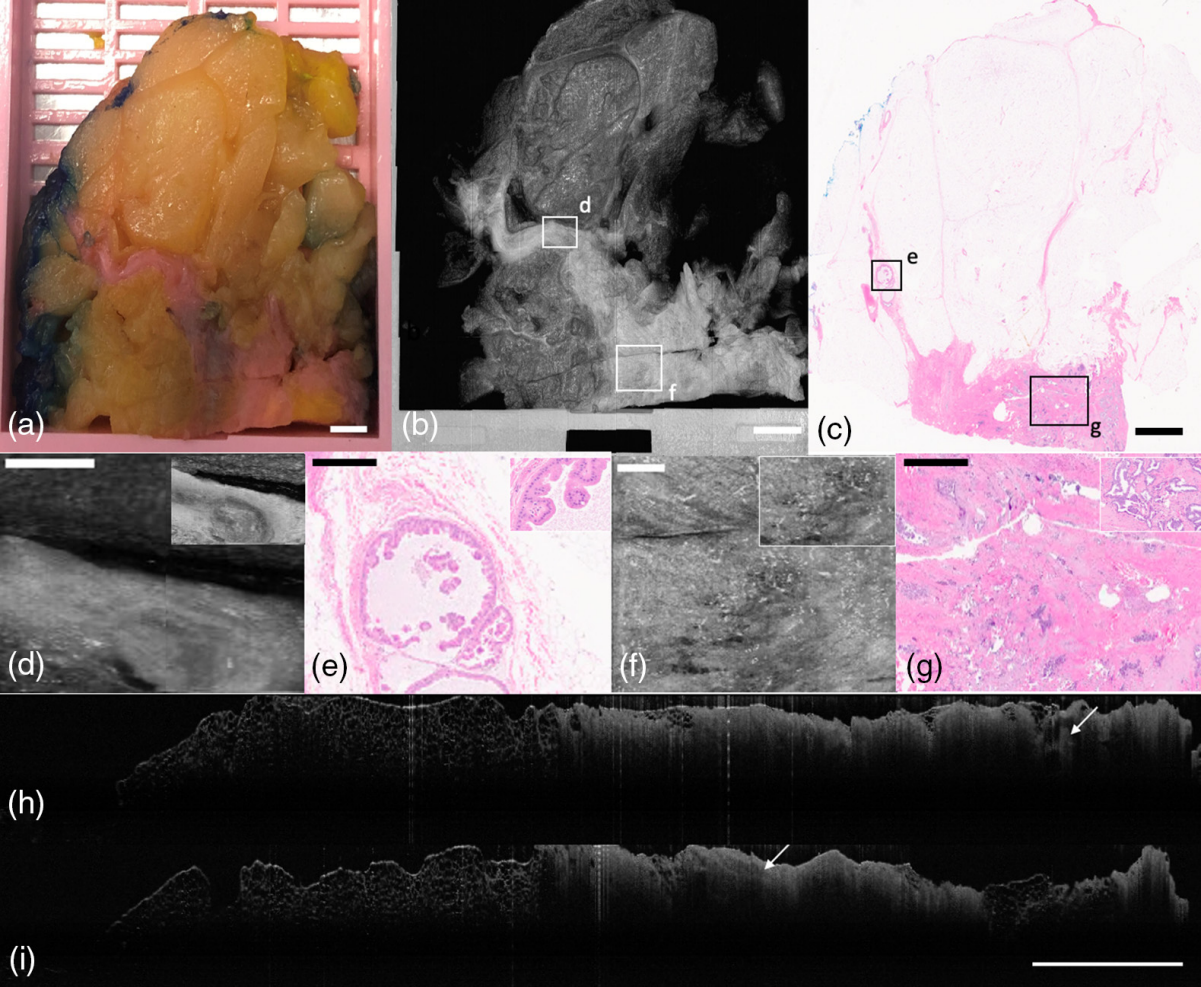
BREAST lumpectomy block showing cystic changes and IDC. (a) Photograph of specimen, (b) standard deviation projection along z-axis, and (c) H&E histology slide of block. (d) OCT *en-face* representation of cystic and papillary apocrine metaplasia; (e) corresponding H&E histology. (f) OCT *en-face* representation of IDC; (g) corresponding H&E histology. (h) OCT B-scan through cystic and papillary apocrine metaplasia (arrow). (i) OCT B-scan through IDC (arrow). (a–c), (h), and (i) Scale bar=3  mm; (d)–(g) 500  μm.

Furthermore, Fig. 5 demonstrates the capacity of OCT to identify nongrossly apparent heterogenous changes in tissue. The section includes non-neoplastic cystic changes, as well as invasive ductal carcinoma (IDC). An enlarged en-face image shows a cystic structure with a clearly defined outer boundary that is typical of cystic and apocrine metaplasia, a non-neoplastic breast lesion [Figs. 5(d) and 5(e)]. An OCT B-scan view of the lesion is shown in Fig. 5(h). Figures 5(f) and 5(g) show that OCT can identify IDC among cystic change, and an OCT B-scan (in a perpendicular plane) is shown in Fig. 5(i).

**Fig. 6:**
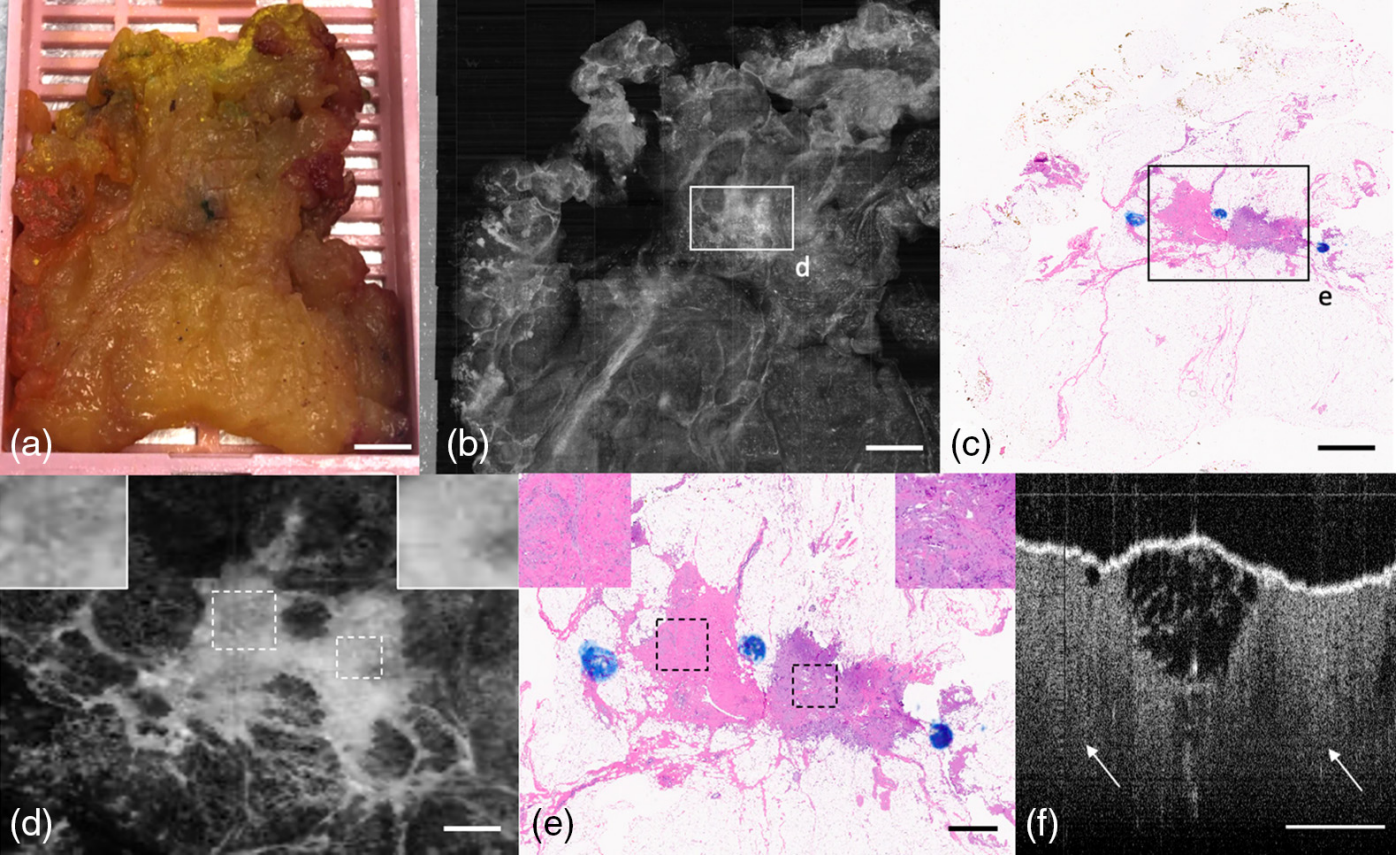
BREAST lumpectomy block diagnosed with focus of invasive carcinoma. (a) Photograph of specimen. (b) Average projection along z-axis. (c) H&E histology slide of block. (d) OCT *en-face* representation of nodule containing reactive stroma with neovascularity and mild chronic inflammation (left) and well-differentiated IDC with reactive stroma (right). Insets show that reactive stroma is more heterogenous and lowly reflective in OCT compared with bright, homogenous cancer. (e) Corresponding H&E histology. (f) OCT B-scan through nodule showing that stroma shows heterogeneous texture and deeper depth penetration (left) compared with highly reflective and lower depth penetration cancer (right). (a)–(c) Scale bar=3  mm; (d)–(f) 500  μm.

In another case, the gross photograph of a tissue block [Fig. 6(a)] shows what appears to be mature adipose tissue, exclusively. OCT and H&E scans of the tissue block [Figs. 6(b) and 6(c)], however, show that, in addition to adipose tissue, there is fibrous tissue admixed with cancer. Figures 6(d) and 6(e) show a higher power *en-face* representation of the admixed tissue, and Fig. 6(f) shows a B-scan through the admixed tissue. This illustrates how OCT achieves near-H&E detection capability of lesions. In Fig. 6(d), it can be observed that the left shows an area with texture that represent neovascularity and mild chronic inflammation. The inset shows the region in more detail, with more homogenous texture and less reflectivity. However, on the right side of the same fibrous area, the tissue appears brighter and more heterogenous with a small interspersed dark spot within the carcinoma. This region corresponds to a well-differentiated desmoplastic invasive carcinoma with reactive stroma interspersed toward the far-right side. Tumors that are desmoplastic (containing a mixture of myofibroblasts, collagen, and neovascularity) reflect stronger than epithelial-rich tumors. The differentiability of these two tissue types is confirmed by the OCT B-scan [Fig. 6(f)], which shows stronger penetration depth on the left side of the fat, and less penetration on the right side.

Figure S4 in the Supplemental Material shows an additional IDC instance.

### 3.2 Imaging Prostate Tissues

**Fig. 7:**
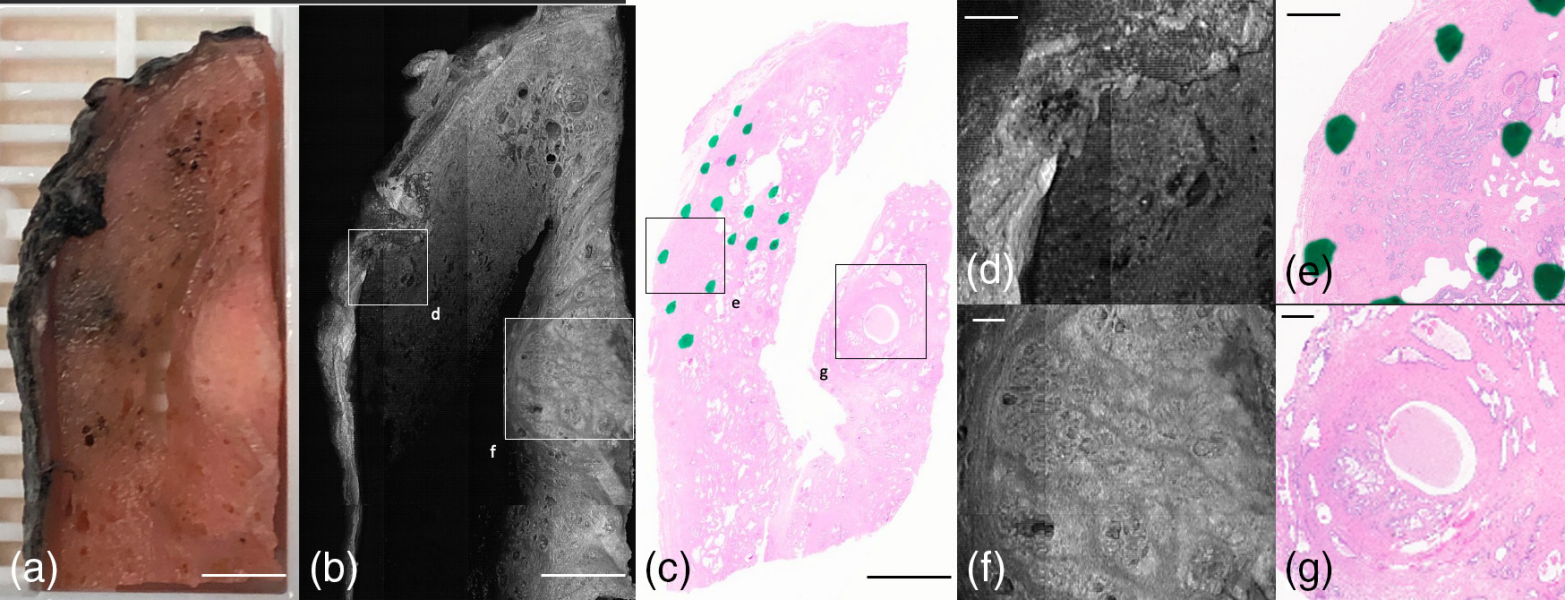
PROSTATE block containing nodular partly cystic BPH, prostatic carcinoma, and fibromuscular stroma. (a) Photograph of specimen. (b) Average projection along z-axis. (c) H&E histology slide of block. (d) OCT *en-face* representation of focus of prostatic carcinoma; (e) corresponding H&E histology. (f) OCT *en-face* representation of nodular BPH; (g) corresponding H&E histology. (a)–(c) Scale bar=3  mm; (d) and (e) 500  μm.

It is well-documented that prostatic carcinoma is difficult to recognize grossly.^20^ Here, we highlight tissues from prostatectomies demonstrating some typical pathologies. Figure 7 shows a block with clearly defined nodular, partly-cystic benign prostatic hyperplasia (BPH). The OCT *en-face* projection shows both macroscopic and microscopic features of the tissue specimen. Although the photograph in Fig. 7(a) shows what appears to be a large white mass, the region of interest is actually on the midleft side of the specimen with a focus of carcinoma [Figs. 7(b) and 7(c)]. Upon further inspection, a focus of cancer is visible in Figs. 7(f) and 7(g), where cancer is surrounded by non-neoplastic prostatic tissue, which is confirmed with the corresponding H&E histology slide.

Given its predominance in prostate tissue, we show further instances of BPH in Figs. S5–S7 in the Supplemental Material.

### 3.3 Imaging Pancreas Tissues

**Fig. 8:**
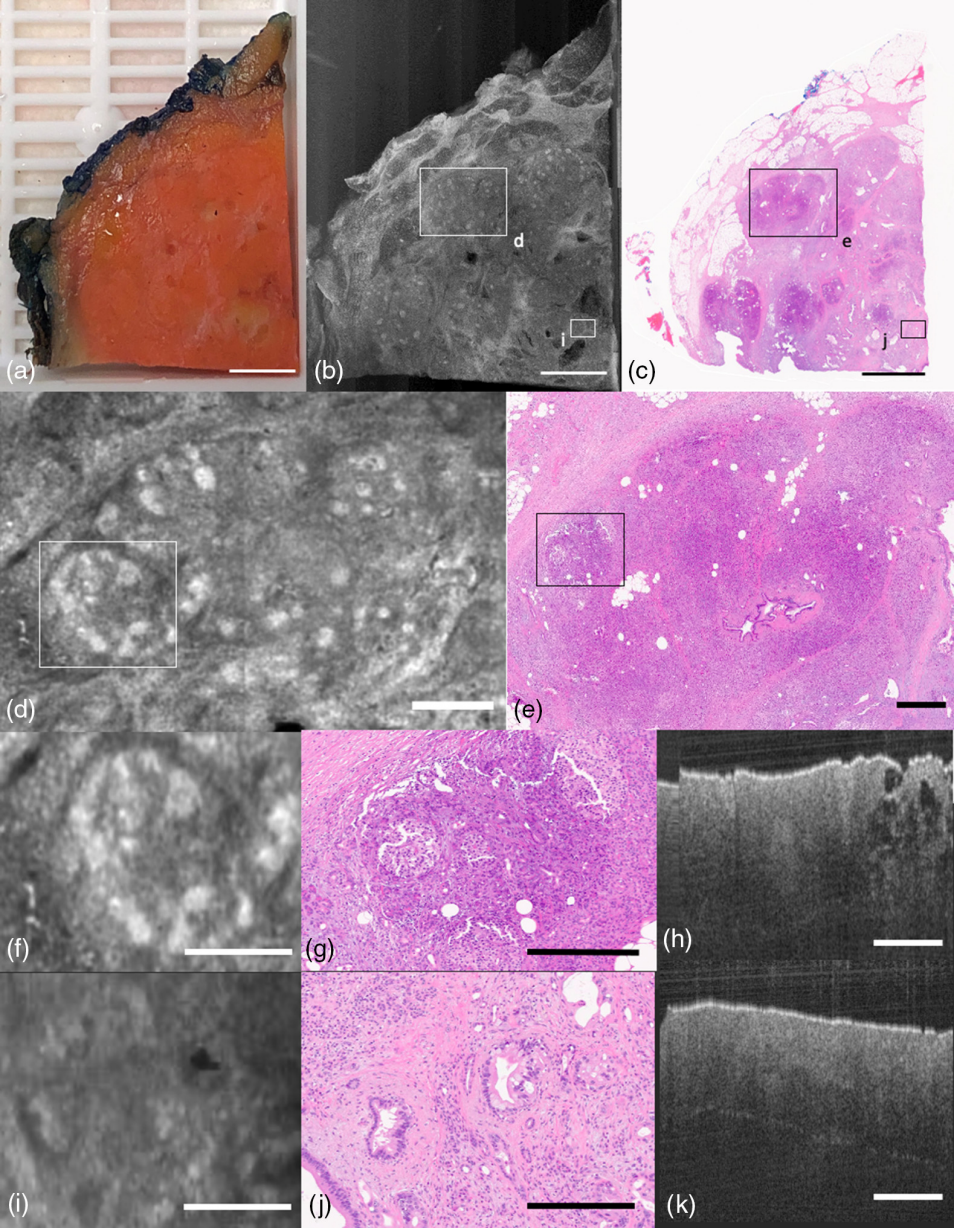
PANCREAS block of pancreatic adenocarcinoma. (a) Photograph of specimen. (b) Average projection along z-axis. (c) H&E histology slide of block. (d) OCT *en-face* representation of normal exocrine pancreas, which represents residual normal pancreas. (e) Corresponding H&E histology. (f) H&E histology of pancreatic acini representation from (d). (g) OCT *en-face* representation of pancreatic acini. (h) OCT B-scan through acini. (i) H&E histology of pancreatic adenocarcinoma glands with a lumen. (j) OCT *en-face* representation of adenocarcinoma. (k) OCT B-scan through carcinoma. (a)–(c) Scale bar=3  mm; (d)–(k) 500  μm.

Gross examination of the pancreas frequently highlights the inability to distinguish between atrophic and inflamed tissue from cancer. A block of pancreatic tissue from a Whipple procedure is shown in Fig. 8. The gross view [Fig. 8(c)] shows pancreatic tissue with a nonclassical lobulated architecture but without a clearly defined mass. Figures 8(d) and 8(e) show non-neoplastic, noninvoluted/residual exocrine pancreas. Figures 8(f)–8(h) show a zoomed-in view of lobulated clusters of non-neoplastic acinar glands. Figures 8(i)–8(k) show glands of pancreatic carcinoma.

### 3.4 Imaging Lung Tissues

**Fig. 9:**
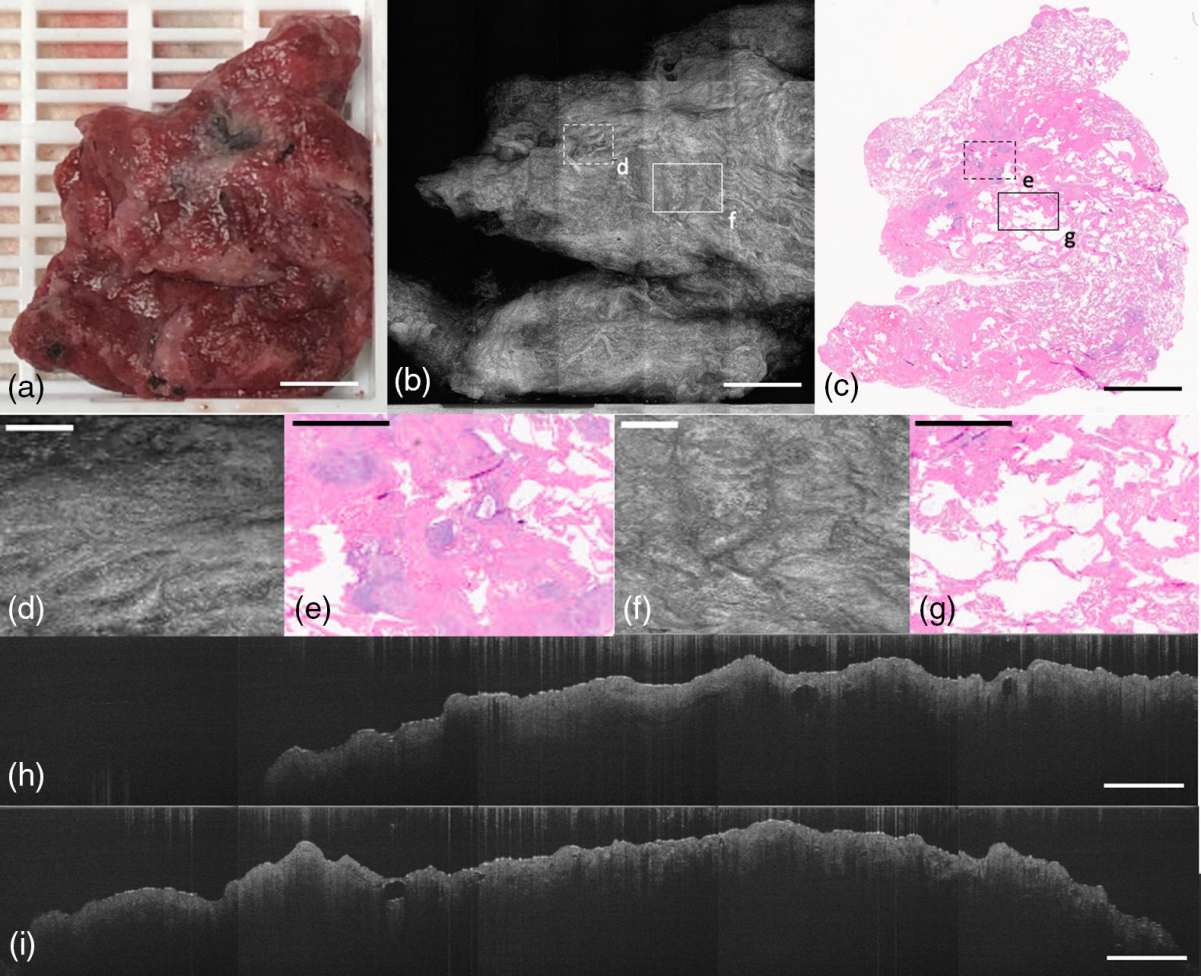
LUNG block with emphysema. (a) Photograph of specimen. (b) Average projection along z-axis. (c) H&E histology slide of block. (d) OCT *en-face* representation of normal fibrotic tissue pancreas; (e) corresponding H&E histology. (f) OCT *en-face* representation of emphysematous tissue; (g) corresponding H&E histology. (h) OCT B-scan of normal fibrotic tissue. (i) OCT B-scan of emphysematous tissue. (a)–(c) Scale bar=3  mm and (d)–(i) 500  μm.

Lung tissue specimens may also demonstrate poor correlation between gross and microscopic evaluation. In Fig. 9, we demonstrate a block of emphysematous tissue from a bilateral lung transplant. The texture of the tissue in the OCT *en-face* projection [Fig. 9(a)] shows the abnormal representation of air spaces within the lung, along with areas of fibrosis. The highlighted area in Figs. 9(d) and 9(e) shows a fibrotic focus, which is more homogeneously textured than the emphysematous component showing multiple cystic spaces in Figs. 9(f) and 9(g). There are also corresponding stitched B-scans in Fig. 9(h) which represent the smooth normal fibrotic tissue, and Fig. 9(i) shows the more heterogenous and lowly reflective emphysematous tissue with very clearly defined textural features.

An example of lung adenocarcinoma is demonstrated in Fig. S8 in the Supplemental Material.

## 4 Discussion and Conclusion

We highlighted a new clinical application and described an OCT system that enables high through-put imaging and fulfills the CAP IVM criteria for *ex vivo* imaging technology. The UHS–OCT system features a 3.7-μm axial resolution, 3.7-μm lateral resolution, 1.1 mm 6-dB sensitivity fall-off range, 10  cm×10  cm field of view, and 250 kHz A-line rate that together enable high-resolution imaging of a pathology cassette in under 1 min. OCT imaging is non-destructive and does not have adverse effects on the tissue; thus it does not interfere with downstream histology, IHC, ISH, or RNA/DNA genomic testing. The field of view matches clinical breast sample sizes, and the resolution enables visualization of cellular-level structures visible in H&E histology.

We imaged with our specially designed OCT system in real-time clinical tissue blocks of a variety of organs, including breast, prostate, pancreas, and lung. We captured OCT images of several typical lesions in each organ. In the breast, we visualized cystic changes, IDC (including desmoplasia), cystic lesions with incipient intraductal papilloma and atypical micropapillary hyperplasia, near-normal breast parenchyma such as fibroadenomatoid hyperplasia, and normal breast parenchyma including ducts, lobules, adipose, stroma, fibrotic tissue, and blood vessels. In the prostate, we captured benign prostatic hyperplasia, prostatic carcinoma, cystically dilated glands, fibromuscular stroma, and dilated glands with corpora amylacea. We visualized normal and diseased pancreas, including normal exocrine pancreas, pancreatic adenocarcinoma, and atrophic and inflamed pancreas. Within the lung, we visualized normal and fibrotic tissue, emphysematous tissue, and adenocarcinoma. The intensity of each tissue structure in an OCT image depends on its absorption and scattering characteristics. Increased intensity (hyperintensity) is due to an increased scattering coefficient, a change in anisotropy coefficient, or a large change in the index of refraction.^21^ Absorption of light (low intensity) is dominated by tissue structures with a low scattering coefficient. For instance, fibrous tissue generally has increased collagen, which increases scattering and thus appears brighter within OCT images.

Real-time histology could be useful for closing a variety of gaps in tissue diagnostics. Currently, gross analysis of excised tissue is dependent upon visual inspection, palpation, and presence of clips or calcifications to identify regions of interest for tissue analysis. Such gross analysis is limited by the variable correlation between macroscopic and microscopic findings. Therefore, imaging systems providing real-time histology should be able to enhance this rather crude and inefficient methodology and allow for microscopic examination of larger volumes of tissue without the need for time-consuming and costly processing. In some organ systems, such as the breast, the discordance between macroscopic and microscopic findings has led to recommendations of complete or near-complete submission of specimens to increase the sensitivity of detection, making the process costly, burdensome, and inefficient. Also, closing this gap may aid in addressing growing histology technician and pathology physician assistant shortages, which have become more acute as specimen volumes increase due to the aging population, increased radiographic sensitivity (at the cost of specificity), and increasing access to healthcare. The low sensitivity of detection of certain types of breast lesions based on gross examination is related to the inherent discordance of the gross and microscopic appearance of these lesions. The absence of a gross signature of many breast lesions resulted in recommendations of extensive sampling to increase the sensitivity of detection of grossly undetected microscopic lesions.^22^

OCT is a promising imaging technology for this application due to its combination of high resolution, fast imaging speed, extended imaging depth in tissue, and lack of need for exogenous contrast agents. The depth of imaging into the tissue allows for a more efficient determination of whether lesions are present given that a typical tissue block is 4-mm thick (may be imaged on both sides to cover the entire thickness of specimen), and is usually only examined histologically by 1 microscopic level by microtoming (occasionally more). Organs with particularly high variability between gross and microscopic findings include breast, prostate, pancreas, and lung. These organs have been previously imaged with OCT for different clinical applications.^23^^–^^26^ OCT imaging of gross tissue from these organs was performed, demonstrating the capacity to identify various organ-typical pathologies that may not be appreciated at the gross level, and confirmed by histology.

Imaging of tissue has been investigated using confocal microscopy,^27^^–^^29^ fluorescence,^30^^–^^35^ nonlinear techniques (including two-photon,^36^ multiphoton,^37^ and stimulated Raman scattering^38^), OCT,^23^ full-field OCT,^39^^–^^41^ optical frequency domain imaging,^37^ microscopy with UV surface excitation,^42^ structured illumination microscopy (SIM),^43^ and light-sheet microscopy.^38^ This nonexhaustive list of cutting-edge research has explored applications relating to rapid intraoperative assessment in place of frozen section analysis,^23^^,^^36^^,^^38^^,^^43^^,^^44^ slide-free pathology,^42^^,^^45^ the detection of cancer in core biopsies,^30^^,^^34^^,^^46^ the selection of tissue for biobanking,^29^ and the study of tumor biology.^37^ Low penetration depth (on the order of a few hundred microns), slow imaging speed, and the need for exogenous contrast that are characteristic of confocal, fluorescence, nonlinear techniques would inhibit the preanalytical issues of pathology analysis. Light sheet microscopy, although capable of achieving ultrafast imaging speed, is limited because it often requires contrast agents or tissue clearing. Preanalytical issues, such as the time of fixation, type of fixation, and/or processing, are considered critical in the standardization of any subsequent pathology analysis including H&E staining, IHC, and/or molecular testing (DNA or RNA). Therefore, any technique modifying these factors is viewed with suspicion. OCT offers a attractive combinatorial technology platform that is well-suited for imaging within the pathology workflow given its high resolution, high speed, and large field of view, which are qualities required for integration into the clinical pathology workflow.

In the future, further advancements can be made to increase the imaging depth and lateral resolution. In addition to these limitations of OCT, others include resolution, field of view, imaging and processing time, and interpretation (specifically who interprets the images and when). We demonstrated that these features facilitate high-quality imaging of cellular-level features at the scale of whole tissue blocks. Further, to overcome the limitations of OCT, combining OCT with other functional imaging modalities such as fluorescent imaging could enhance the utility.^47^

Our previous works on OCT classification of breast tissues utilizing machine learning^48^^,^^49^ and deep learning^50^ algorithms give us confidence that we can apply similar approaches to identify the above-illustrated lesions across organs.^51^ This approach and its associated speed suggest that these algorithms could be efficiently integrated into the clinical workflow. Many quantitative approaches have also been investigated for tissue differentiation.^51^ We are mindful that such classification schemes require a substantial database of annotated images, which we hope to acquire in the future.

Looking toward the future, we envision (1) an efficient system to determine regions of non-interest (an easier task than identifying interest) in cases of total or near-total tissue submission to eliminate noncontributory tissue sections, (2) identifying positively a variety of histologic lesions for subsequent analysis, (3) in addition to the analysis of blocks destined for tissue processing and histologic examination, analyzing tissue slices, especially in cases in which the whole specimen is not submitted to determine positively regions of interest, (4) that such interpretation will occur by implementing an AI algorithm, (5) that such approaches can be utilized to enhance all phases of the diagnostic process (biopsy, quality assurance, intraoperative margin determination, frozen section, tumor banking, at time of tissue grossing), and (6) that these approaches can be applied to a variety of organ/tissue systems.

In summary, OCT-based real-time histology evaluation in clinical settings promises to address a heretofore unaddressed gap that has been present for more than 100 years and thereby enhance and transform the gross pathology diagnostic process across the diagnostic continuum and across organs.
