# Supplementary material for: Optical coherence tomography holds promise to transform the diagnostic anatomic pathology gross evaluation process
Source: J Biomed Opt. 2022 Sep 1;27(9):096003. doi: 10.1117/1.JBO.27.9.096003 (PMC9434002; doi:10.1117/1.JBO.27.9.096003)
Supplement: Supplementary file 1 [file JBO_027_096003_SD001.pdf]

Optical coherence tomography (OCT) holds promise to transform the diagnostic anatomic pathology gross evaluation process

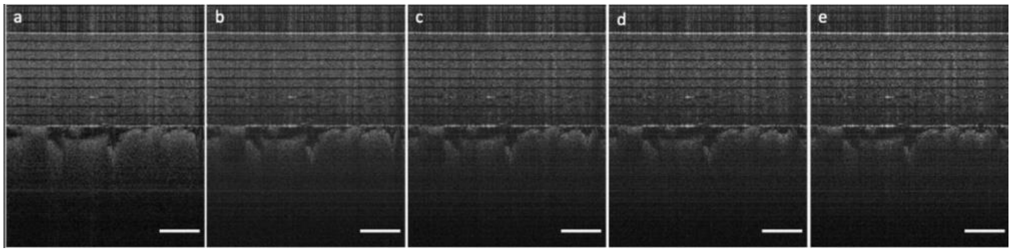

Fig. S1. Imaging performance as a function of A-line rate. Representative single B-scan images of 10 layers of tape on corkboard at increasing A-line rates **(a)** 50 kHz **(b)** 100 kHz **(c)** 150 kHz **(d)** 200 kHz **(e)** 250 kHz. The saturation level of the dynamic range in the spectral shaping post-processing is reduced by 10% with each 50 kHz increase in A-line rate to account for the reduction in photons detected at faster imaging speeds. Scale bar = 500 µm.

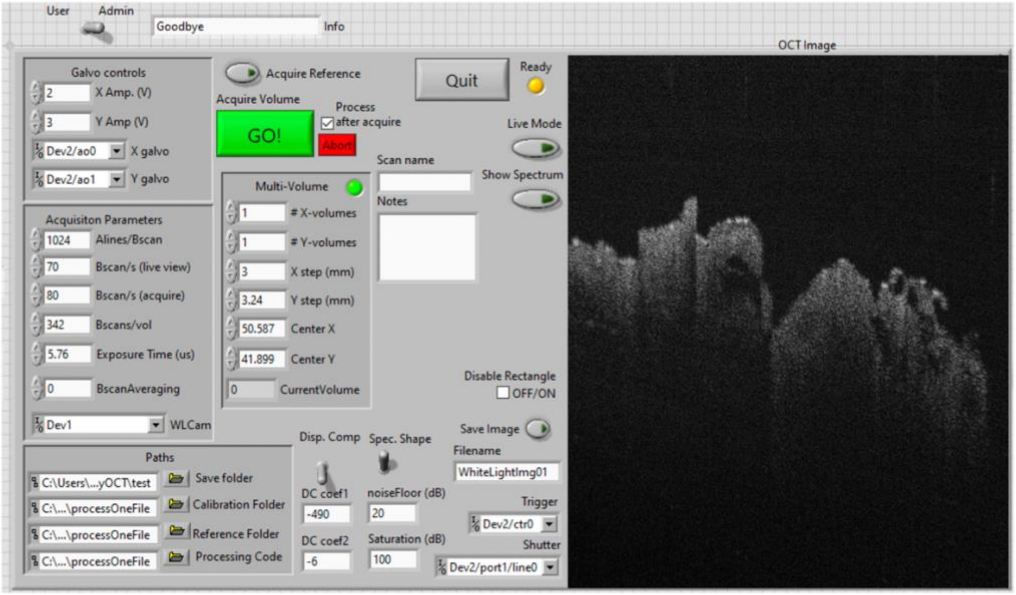

Figure S2. UHS-OCT image acquisition graphical user interface (GUI) designed in LabView. Easy to use interface allows for user to adjust the galvanometer controls, camera acquisition parameters, post-processing options (spectral shaping and dispersion compensation), and initiate multi-volume acquisition. The live OCT display provides real-time imaging feedback by utilizing the optimized C image reconstruction algorithm to rapidly process and display images.

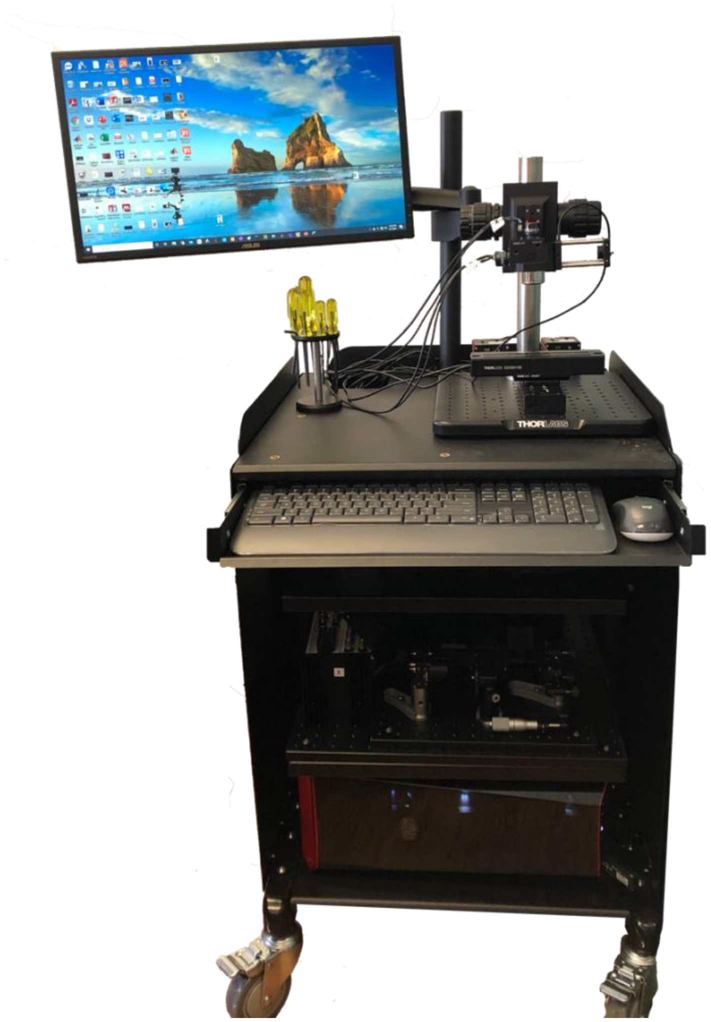

Figure S3. Photograph of UHS-OCT system mounted in a medical device cart. The sample arm scanner is on the top surface of the cart, with optical components on the shelf below, along with electronic controllers, and the computer on the bottom shelf.

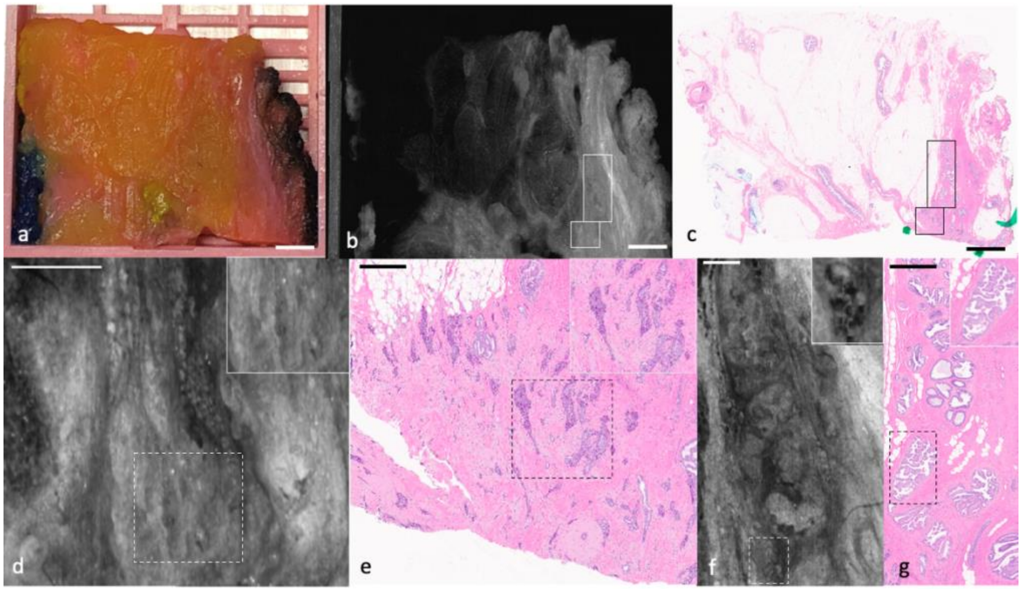

Figure S4. Breast lumpectomy block with a focus of invasive ductal carcinoma. (**a**) Photograph of specimen (**b**) Average OCT en-face projection along z-axis (**c**) H&E histology slide of block (**d**) OCT en-face representation of invasive ductal carcinoma (**e**) Corresponding H&E histology (**f**) OCT en-face representation of cystic lesion with incipient intraductal papilloma and atypical micropapillary hyperplasia (**g**) Corresponding H&E histology. Scale bar = 3mm (a-c); 500µm (d-g).

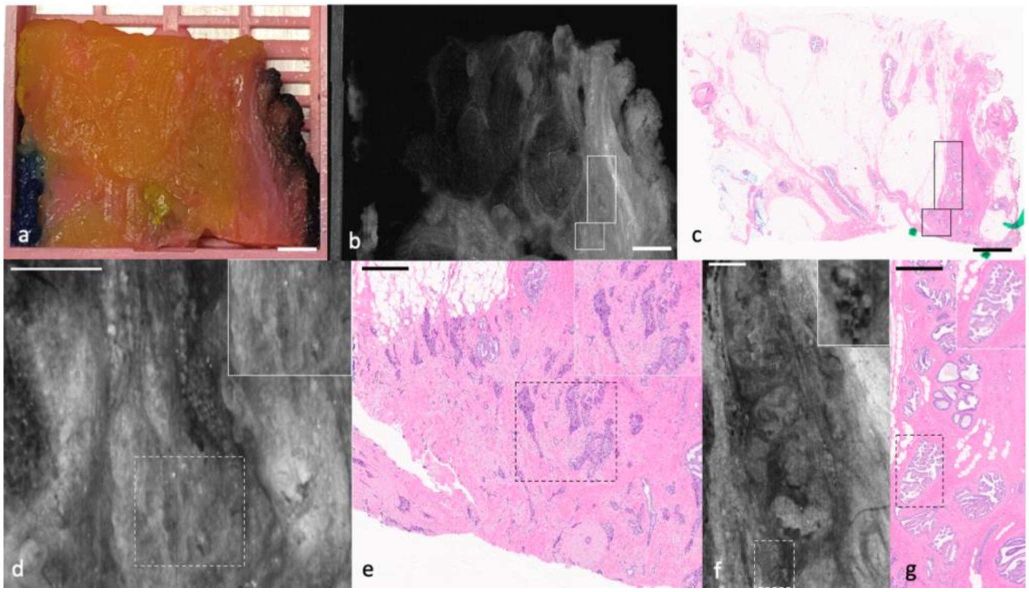

Figure S5. Breast lumpectomy block with a focus of invasive ductal carcinoma. (**a**) Photograph of specimen (**b**) Average OCT en-face projection along z-axis (**c**) H&E histology slide of block (**d**) OCT en-face representation of invasive ductal carcinoma (**e**) Corresponding H&E histology (**f**) OCT en-face representation of cystic lesion with incipient intraductal papilloma and atypical micropapillary hyperplasia (**g**) Corresponding H&E histology. Scale bar = 3mm (a-c); 500µm (d-g).

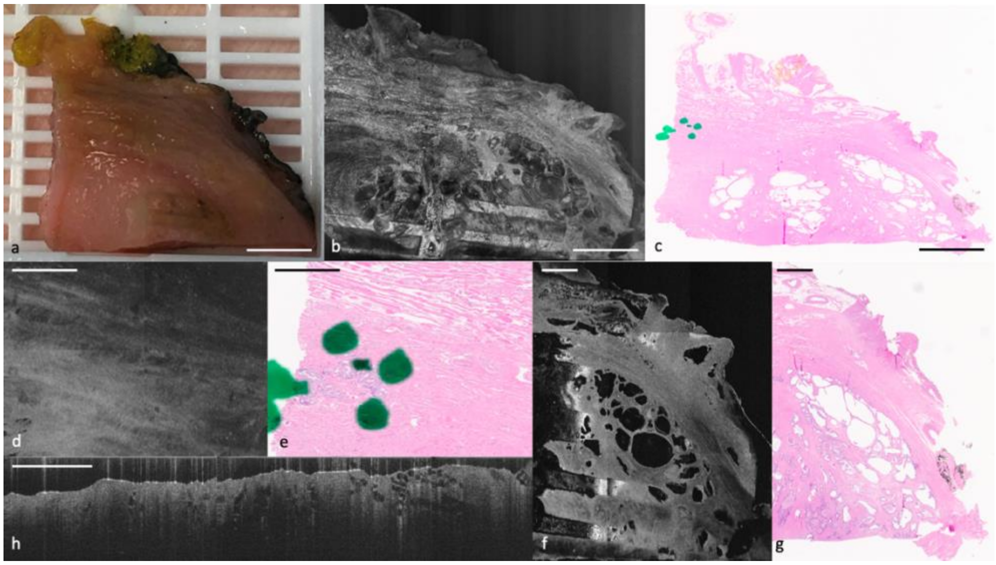

Figure S6. Prostatectomy block demonstrating benign prostatic hyperplasia (BPH). (**a**) Average projection along z-axis. (**b**) H&E histology slide of block (**c**) Photograph of specimen (**d**) OCT en-face representation of minute focus of prostatic carcinoma (**e**) Corresponding H&E histology. Green dots correspond to pathologist marking for clinical diagnosis (**f**) OCT en-face representation of cystically dilated glands (**g**) Corresponding H&E histology (**h**) Single OCT B-scan through fibrous area of tissue shows fat content at the periphery of the specimen. Scale bar = 3mm (a-c); 500µm (d-h).

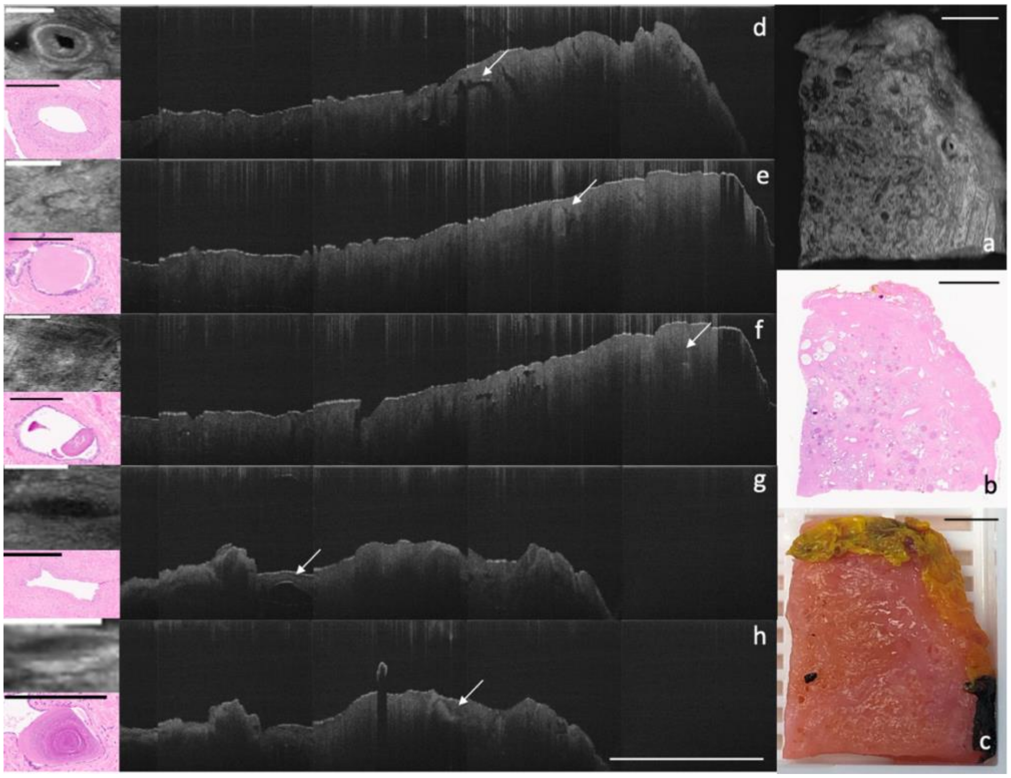

Figure S7. Prostatectomy block demonstrating benign prostatic hyperplasia (BPH). (a) Average projection along z-axis **(b)** H&E histology slide of block **(c)** Photograph of specimen **(d)** OCT B-scan containing blood vessel. Insets show OCT en-face of blood vessel and corresponding H&E **(e)** OCT B-scan containing corpora amylacea. Insets show OCT en-face of corpora amylacea and corresponding H&E **(f)** OCT B-scan containing mild dilation of gland with corpora amylacea. Insets show OCT en-face of gland with amylacea and corresponding H&E **(g)** OCT B-scan containing blood vessel. Insets show OCT en-face of vessel and corresponding H&E **(h)** Single OCT B-scan containing classic case of corpora amylacea. Insets show OCT en-face of corpora amylacea and corresponding H&E. Scale bar = 3mm (a-h); 500µm (insets).

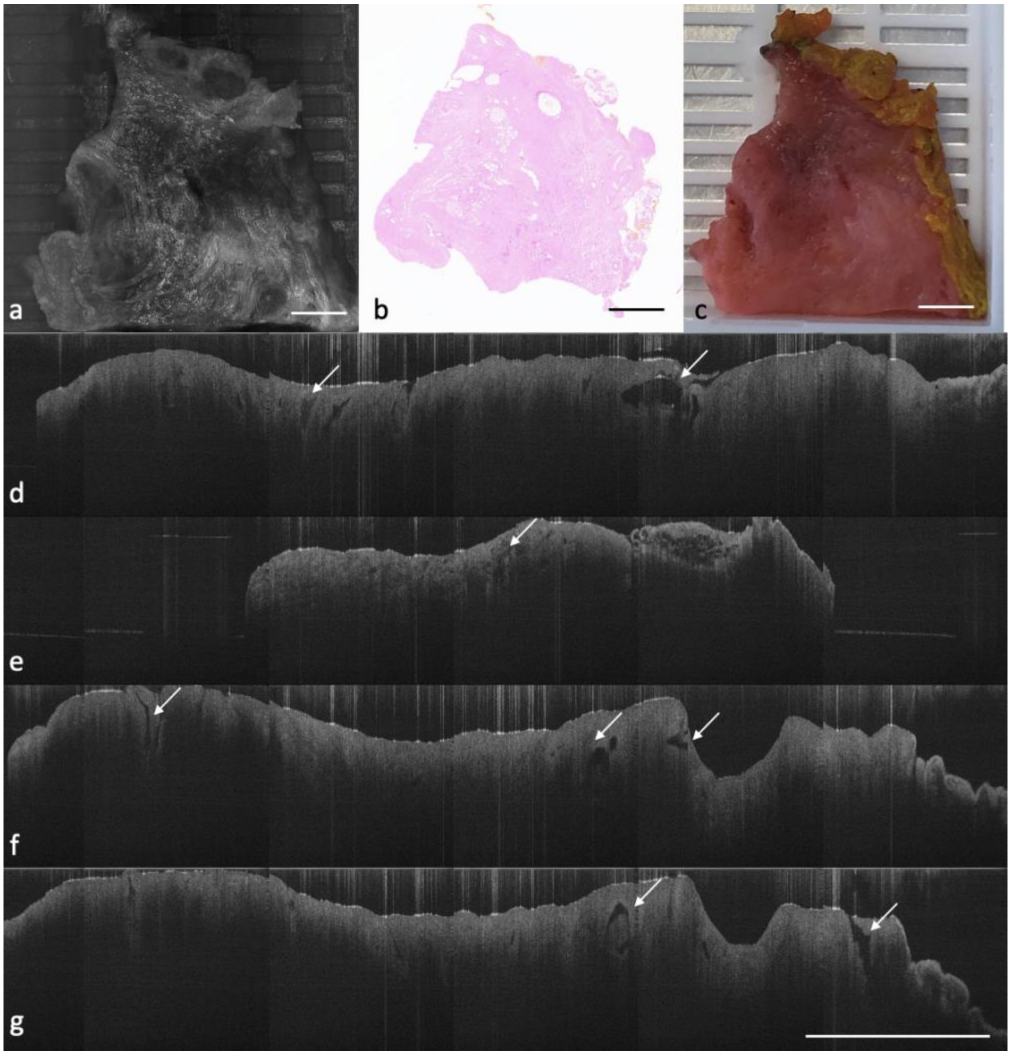

Figure S8. Prostatectomy block containing benign prostatic hyperplasia (BPH) with gland predominance. (a) Average projection along z-axis **(b)** H&E histology slide of block **(c)** Photograph of specimen **(d-g)** Representative single OCT B-scans containing characteristic features of BPH (arrows). Scale bar = 3mm.

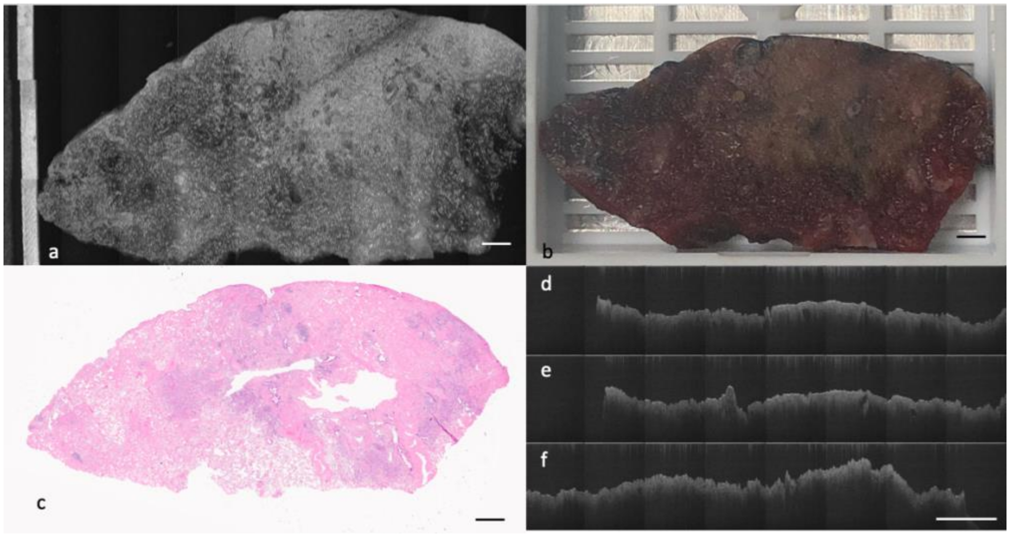

Figure S9. Lobectomy block of lung adenocarcinoma. (a) Average projection along z-axis **(b)** H&E histology slide of block **(c)** Photograph of specimen **(d-f)** Representative single OCT B-scans show that the adenocarcinoma is strongly attenuating and thus it can be observed that the penetration depth is shallow in regions of cancer. The adenocarcinoma has a characteristic lepidic pattern (lining alveolar spaces), and occupies a substantial portion of the tissue shown, particularly on the right side. The central portion of this mass is fibrotic scar tissue with tumor surrounding it. The vertical lines represent sequentially stitched B-scans. Scale bar = 3mm (a-c); 500µm (d-f).
